# A role for the *Saccharomyces cerevisiae* Rtt109 histone acetyltransferase in R-loop homeostasis and associated genome instability

**DOI:** 10.1093/genetics/iyac108

**Published:** 2022-07-22

**Authors:** Juan Carlos Cañas, María Luisa García-Rubio, Alicia García, Francisco Antequera, Belén Gómez-González, Andrés Aguilera

**Affiliations:** Centro Andaluz de Biología Molecular y Medicina Regenerativa-CABIMER, Universidad de Sevilla-CSIC, 41092 Seville, Spain; Departamento de Genética, Facultad de Biología, Universidad de Sevilla, 41012 Seville, Spain; Centro Andaluz de Biología Molecular y Medicina Regenerativa-CABIMER, Universidad de Sevilla-CSIC, 41092 Seville, Spain; Departamento de Genética, Facultad de Biología, Universidad de Sevilla, 41012 Seville, Spain; Instituto de Biología Funcional y Genómica (IBFG), CSIC-Universidad de Salamanca, 37007 Salamanca, Spain; Instituto de Biología Funcional y Genómica (IBFG), CSIC-Universidad de Salamanca, 37007 Salamanca, Spain; Centro Andaluz de Biología Molecular y Medicina Regenerativa-CABIMER, Universidad de Sevilla-CSIC, 41092 Seville, Spain; Departamento de Genética, Facultad de Biología, Universidad de Sevilla, 41012 Seville, Spain; Centro Andaluz de Biología Molecular y Medicina Regenerativa-CABIMER, Universidad de Sevilla-CSIC, 41092 Seville, Spain; Departamento de Genética, Facultad de Biología, Universidad de Sevilla, 41012 Seville, Spain

**Keywords:** R-loops, DNA–RNA hybrids, histone acetylation, sister-chromatid recombination, genetic instability

## Abstract

The stability of the genome is occasionally challenged by the formation of DNA–RNA hybrids and R-loops, which can be influenced by the chromatin context. This is mainly due to the fact that DNA–RNA hybrids hamper the progression of replication forks, leading to fork stalling and, ultimately, DNA breaks. Through a specific screening of chromatin modifiers performed in the yeast *Saccharomyces cerevisiae*, we have found that the Rtt109 histone acetyltransferase is involved in several steps of R-loop-metabolism and their associated genetic instability. On the one hand, Rtt109 prevents DNA–RNA hybridization by the acetylation of histone H3 lysines 14 and 23 and, on the other hand, it is involved in the repair of replication-born DNA breaks, such as those that can be caused by R-loops, by acetylating lysines 14 and 56. In addition, Rtt109 loss renders cells highly sensitive to replication stress in combination with R-loop-accumulating THO-complex mutants. Our data evidence that the chromatin context simultaneously influences the occurrence of DNA–RNA hybrid-associated DNA damage and its repair, adding complexity to the source of R-loop-associated genetic instability.

## Teaser

Genome stability is threatened by DNA-RNA hybrids and R loops. Cañas et al. find the Rtt109 histone acetyltransferase prevents DNA-RNA hybridization by targeting histone H3 lysines 14 and 23. By acetylating lysines 14 and 56, Rtt109 functions in the repair of replication-born DNA breaks, such as those ultimately caused by R-loops. These findings reveal a key role of chromatin in both the occurrence of spontaneous DNA damage and its repair, adding complexity to R loop-associated genetic instability.

## Introduction

DNA–RNA hybrids and R-loops, formed by the hybrid and the displaced single-strand DNA are sources of genetic instability (reviewed in Garcia-Muse and Aguilera 2019). Due to their potential harmfulness, a vast amount of cellular proteins have evolved to prevent DNA–RNA hybrid formation or to resolve them. These proteins include cotranscriptional RNA-binding factors, such as the THO complex (Huertas and Aguilera 2003; Luna *et al.* 2019), which protect the nascent RNA from hybridizing back with its DNA template, and the DNA–RNA UAP56/DDX39B (yeast Sub2) helicase that unwind hybrids during transcription elongation (Perez-Calero *et al.* 2020). Multiple other helicases can also unwind hybrids although they can act in different cell cycle moments, such as senataxin (Mischo *et al.* 2011; Skourti-Stathaki *et al.* 2011), whose loss causes hybrid accumulation in yeast S-phase, likely as a consequence of head-on transcription-replication conflicts (San Martin-Alonso *et al.* 2021). In addition, if formed, the RNA moiety of DNA–RNA hybrids can be degraded by type H ribonucleases, like RNase H1 (Cerritelli and Crouch 2009). DNA–RNA hybrids are significantly enriched upon depletion or inactivation of any of these factors. Evidence from yeast to human cells supports that DNA–RNA hybrids challenge genome integrity mainly by interfering with the progression of replication forks (reviewed in Gomez-Gonzalez and Aguilera 2019). As a result of replication fork progression impairment, R-loop accumulating THO mutants rely on checkpoint factors such as the clamp loader Rad24 or the 9-1-1 complex (Rad17/Mec3/Ddc1) for survival, particularly in the presence of replicative stress (Gomez-Gonzalez *et al.* 2009).

The formation and resolution of R-loops and their associated genetic instability are also influenced by the chromatin context. Along this line, it has been shown that the depletion of the FACT complex or histone H1 gives rise to the accumulation of hybrids and replication stress (Herrera-Moyano *et al.* 2014; Bayona-Feliu *et al.* 2017; Almeida *et al.* 2018) and that the SWI/SNF remodeling complex helps resolve R-loop-mediated transcription-replication conflicts (Bayona-Feliu *et al.* 2021). Furthermore, DNA–RNA hybridization can be modulated by histone post-translational modifications (PTMs). The inhibition of histone deacetylation by chemical compounds or by the depletion of the human SIN3A histone deacetylase complex has been reported to cause R-loop accumulation likely due to the more open chromatin state conferred by hyperacetylation (Salas-Armenteros *et al.* 2017). Similarly, *sin3Δ* yeast mutants were reported to accumulate R-loops (Wahba *et al.* 2011). In addition, certain histone N-terminal tail mutations stimulate the formation of DNA–RNA hybrids, likely due to their inability to be modified, although neither the histone modifiers nor the PTMs involved are known. These mutations include the deletion of the 28 first amino acids of histone H3 (H3Δ1-28 mutant) or the changes of 4 of its H3 lysines (K9, K14, K18, and K23) to alanines (H3K9-23A mutant; Garcia-Pichardo *et al.* 2017).

To further investigate the role of chromatin modifications in R-loop homeostasis, we assayed DNA–RNA hybrid levels in a selection of null mutants of known histone modifiers, including deacetylases, demethylases, acetyltransferases, and methyltransferases. Loss of Rtt109 histone acetyltransferase increases the cellular levels of R-loops. Our data support that this phenotype is caused by the lack of H3K14 and H3K23 acetylation and provide new perspective to discuss the role of Rtt109 in the maintenance of genetic stability associated to DNA–RNA hybrids.

## Materials and methods

### Yeast strains and plasmids

Yeast strains and plasmids used in this study are indicated in Supplementary Tables 1 and 2, respectively.

### S9.6 immunofluorescence of yeast chromosome spreads

Chromosome spreads were performed as described in Chan *et al.* (2014) with some modifications. 10 mL of exponential cultures were pelleted and washed in cold spheroplasting buffer (1.2 M Sorbitol, 0.1 M potassium phosphate, 0.5 MgCl_2_) and then digested by adding the same buffer with 10 mM DTT and 3 mg/mL Zymolyase 20T and incubating at 37°C during 10 min. Digestion was stopped by adding 2 volumes of solution 2 (0.1 M MES, 1 M sorbitol, 1 mM EDTA, 0.5 mM MgCl_2_ pH 6.4). Spheroplasts were centrifuged and transferred onto slides where they were lysed with 1% lipsol and fixed with fixative solution (4% paraformaldehyde, 3.4% sucrose). The spreading was carried out using a glass rod and the slides were dried overnight in the extraction hood.

Slides were then washed in 1× PBS during 30 min and incubated in blocking buffer (5% BSA, 0.2% milk in 1× PBS) during 10 min in humid chambers. Afterwards, slides were incubated with the S9.6 monoclonal antibody (hybridoma cell line HB-8730) diluted 1:300 in blocking buffer at 23°C during 1 h. Slides were washed 3 times in 1× PBS and incubated with secondary Cy3 conjugated goat antimouse (Jackson laboratories, #115-165-003) diluted 1:1,000 in blocking buffer at room temperature during 1 h. Finally, slides were washed in 1X PBS and mounted with 50 µL of Vectashield (Vector laboratories) with 1× DAPI and sealed with nail polish. More than 150 nuclei were visualized and counted in a fluorescence microscopy Leica DC 350F microscope to obtain the fraction of nuclei with detectable DNA–RNA hybrids.

### DNA–RNA hybrid immunoprecipitation assays

DNA–RNA hybrid immunoprecipitation (DRIP) experiments were performed as described. Cells coming from exponential growth were pelleted by centrifugation and resuspended in 2.4 mL of spheroplasting buffer (1 M sorbitol, 2 mM Tris–HCl pH 8.0, 100 mM EDTA pH 8.0, 0.1% v/v β-mercaptoethanol, 2 mg/mL Zymoliase 20T). Samples were incubated at 30°C during 30 min. After centrifugation, pellet was resuspended in 1.125 mL of buffer G2 (0.8 mM Guanidine HCl, 30 mM Tris–HCl pH 8.0, 30 mM EDTA pH 8.0, 5% Tween 20, 0.5% Triton X-100) together with 40 µL of 10 mg/mL RNase A and incubated at 37°C during 30 min. Then, 75 µL of 20 mg/mL proteinase K were added and samples stood at 50°C for 1 h. DNA was purified by chloroform: isoamyl alcohol (24:1) and precipitated with 1 volume of isopropanol. With the help of a glass Pasteur pipette, DNA was transferred to a new eppendorf where resuspended in 150 µL of 1× TE (1 mM Tris-HCl pH 7.5, 0.5 mM EDTA pH 8.0) and digested overnight with 50 U of HindIII, EcoRI, BsrGI, XbaI, and SspI. Half of the DNA was treated with 8 µL of RNase H (New England BioLabs) overnight at 37°C as RNase H control. One hundred and thirty microliters from each sample were incubated for immunoprecipitation (IP) with S9.6 antibody-Dynabeads Protein A (Invitrogen) complexes (previously incubated overnight at 4°C) during 2.5 h at 4°C. The remaining 20 µL were incubated without the S9.6 antibody for the determination of the input levels. Samples were then washed 3 times with 1× binding buffer (10 mM NaPO_4_ pH 7.0, 0.14 M NaCl, 0.05% Triton X-100). DNA was eluted in 100-µL elution buffer (50 mM Tris pH 8.0, 10 mM EDTA, 0.5% SDS) treated 45 min with 7 µL of 20 mg/mL proteinase K at 55°C and purified with Macherey-Nagel DNA purification kit. Real-time quantitative PCR was performed using iTaq universal SYBR Green (Biorad) with a 7500 Real-Time PCR machine (Applied Biosystems). Signals obtained from the IP were divided by signals obtained from the input to estimate the percentage input and then normalized to the percentage input obtained in the wild-type untreated control sample. Primers sequences used for this analysis were: GTCAGAGGCTATATTTCACTGGAGA and TACGTCTTGTTTCGGCCTTAATC for *PDR5*, CCCGTGGTAAACCTTTAGAAA and ATATGAACGGCAAATTGAGAC for *SPF1*, TTGTGCCCGAATCCAGTGA and TGGCGGCTTCAGTGTTTCTA for *GCN4*, and CCTTGATACGAGCGTAACCATCA and GAAGGTATGAGATGGGCTGGTAA for *PDC1*.

### Recombination assays

Transformants with the recombination system were plated on glucose- or galactose-containing media and grown at 30°C during 3–4 days. Appropriate dilutions of cultures from 6 independent colonies were plated on YPD or media lacking leucine to count for total or recombinant cells, respectively. Recombination frequencies were calculated as previously described as means of at least 3 median frequencies obtained each from 6 independent colonies isolated in the appropriate medium for the selection of the required plasmids (Gomez-Gonzalez *et al.* 2011).

### Rad52 foci detection

Yeast strains were transformed with pWJ1344 or pWJ1213 plasmids (Lisby *et al.* 2001). Resulting transformants were grown in glucose- or galactose-containing selective media until exponential growth and cells were then fixed with 2.5% formaldehyde in 0.1 M KHPO pH 6.4 during 10 min followed by 2 washes in 0.1 M KHPO pH 6.6. Afterwards, cells were washed in 0.1 M KHPO pH 7.4 and permeabilized with 80% ethanol during 10 min, followed by resuspension in 1 µg/mL DAPI for staining nuclei. More than 200 nuclei for each experiment were visualized and counted in a fluorescence microscopy Leica DC 350F microscope.

### Nucleosome analysis by MNase-seq and DANPOS

Mononucleosomal DNA was isolated as described previously (Gonzalez *et al.* 2016). Briefly, *Saccharomyces cerevisiae* cultures of 200 mL at 0.8 × 10^7^ cells/mL were collected for the preparation of mononucleosomal DNA. Cells were fixed with 1% formaldehyde and treated with 10 mg of Zymolyase 20T for 5 min at 30°C to generate spheroplasts. These were resuspended in NP-buffer (1 M Sorbitol; 50 mM NaCl; 10 mM Tris pH7.4; 5 mM MgCl_2_; 1 mM CaCl_2_; 0.075% NP-40; 1 mM β-Mercaptoetanol; 500 μM Spermidine) and digested with 300 units/mL of micrococcal nuclease (MNase) at 37°C during 10 min. The amount of MNase was optimized experimentally for each strain to generate an 80:20 ratio of mononucleosome to dinucleosome fragments. Mononucleosomal DNA fragments were recovered from 1.5% agarose gels.

Libraries of mononucleosomal DNA were constructed following the Illumina protocol and were sequenced in an Illumina NextSeq500 platform using the paired-end protocol. We generated between 13 and 32 million reads of 75 nt per experiment, representing an 83- to 203-fold genome coverage. Reads were aligned using Bowtie (Langmead *et al.* 2009) to the *S. cerevisiae* SacCer 3 genome version. Alignment files were processed using the NucWave algorithm (Quintales *et al.* 2015) to generate the nucleosome occupancy maps. We analyzed biological duplicates of MNase-seq maps of all the strains described in the text.

The DANPOS 2 application (Chen *et al.* 2013) was used to calculate the difference in nucleosome fuzziness at genome-wide scale using the dpos utility with a span of 1 bp and a read extension of 50 bp to make it compatible with NucWave maps. We considered significant the level of log2-fold change of fuzziness for each strain relative to its control when the mean of the difference between the 2 genome-wide maps was above 2σ (σ = 0.1), with an enrichment *P*-value < 0,05 based on Fisher’s exact test.

### Sister-chromatid recombination analysis

Analysis of the double-strand breaks (DSB) and sister-chromatid recombination (SCR) intermediates was performed essentially as described in Ortega *et al.* (2019) and Gomez-Gonzalez *et al.* (2011). Cells transformed with pTHGH plasmid were grown in SRaf lacking tryptophan for plasmid selection and in the presence of 5 µg/mL doxycycline in order to repress *LEU2* gene expression until exponential growth. Galactose at 2% (final concentration) was added to induce HO endonuclease overexpression and samples were collected after indicated time points. DNA was extracted by phenol-chloroform-isoamyl alcohol (25:24:1) and precipitated in isopropanol. DNA was resuspended in 200 µL of 1× TE (1 mM Tris-HCl pH 7.5, 0.5 mM EDTA pH 8.0) and digested with SpeI and XhoI overnight. DNA was precipitated in isopropanol and samples were electrophoresed using a 0.8% agarose gel. Finally, DNA was transferred into a Hybond N (GE Healthcare) membrane and hybridized with ^32^P-labeled 0.22 Kb *LEU2* probe. Quantification was performed by dividing the signal of the bands corresponding to DSBs (2.4- and 1.4-kb bands) or SCR-specific intermediates (4.7-kb band) by the signal of the total DNA in each line (sum of the signals in all the bands). The 2.9-kb band was not used for SCR quantification as it can also arise by another non-SCR intermediates such as intrachromatid recombination [see Ortega *et al.* (2019) and Gomez-Gonzalez *et al.* (2011) for more details]. We then normalized all signals to the time point 0 by subtracting the signal obtained at this time point in which there is no DSB formation.

## Results

### Rtt109 histone acetyltransferase regulates DNA–RNA hybrid homeostasis

**Fig. 1.**
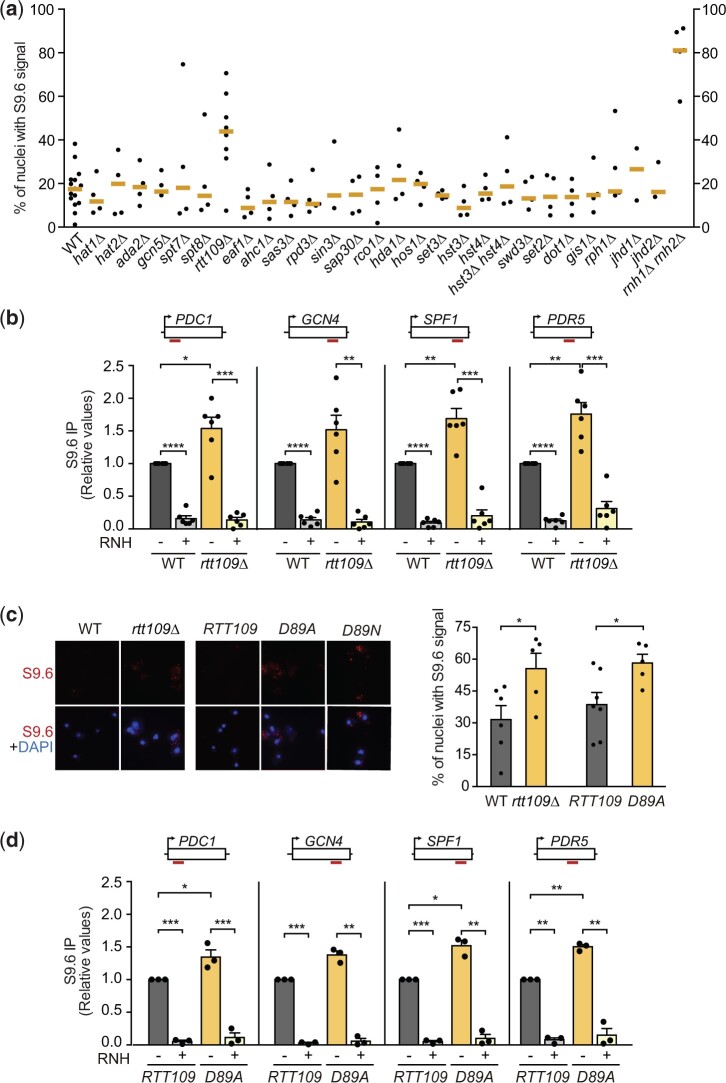
*rtt109Δ* leads to R-loop accumulation. a) Percentage of nuclei with S9.6 signal as detected by IF on chromosome spreads in the indicated strains. The median value of several independent experiments, each performed with at least 150 cells, is plotted. b) DRIP using S9.6 antibody in the indicated regions of the genome, as detected by qPCR in WT and *rtt109Δ* cells. A scheme of the region analyzed is shown above each graph. Average and SEM of 6 independent experiments are shown. *P < 0.05, **P < 0.01, ***P < 0.001, ****P < 0.0001 (paired Student’s *t*-test). c) Percentage of nuclei with S9.6 signal in the indicated strains complemented with an empty vector, a vector with the wild-type *RTT109* gene or with *rtt109-D89A* mutant. Average and SEM of 5 or 6 independent experiments and representative images are shown. * *P* < 0.05 (unpaired Student’s *t*-test). d) DRIP in the indicated regions of the genome, as detected by qPCR in WT and *rtt109Δ* transformed with an empty vector, a vector with the wild-type *RTT109* gene or with *rtt109-D89A* mutant. A scheme of the region analyzed is shown above each graph. Average and SEM of 3 independent experiments are shown. *P < 0.05, **P < 0.01 (paired Student’s *t*-test).

To search for histone modifiers with a role in DNA–RNA hybrid homeostasis, we performed a specific characterization of viable deletion mutants available in the Euroscarf collection by immunofluorescence (IF) with the anti-DNA–RNA hybrid S9.6 antibody. As a positive control strain accumulating high DNA–RNA hybrid levels, we used the *rnh1Δ rnh201Δ* double mutant, in which 81% of the nuclei showed S9.6 signal (Fig. 1a). We analyzed null mutants of histone acetyltransferases (HAT; *hat1Δ, hat2Δ, ada2Δ, gcn5Δ, spt7Δ, spt8Δ, rtt109Δ, eaf1Δ, ahc1Δ, sas3Δ*), histone deacetylases (HDAC; *rpd3Δ, sin3Δ, sap30Δ, rco1Δ, hda1Δ, hos1Δ, set3Δ, hst3, hst4Δ*), histone methyltransferases (*swd3Δ, set2Δ, dot1Δ*), and histone demethylases (*gis1Δ, rph1Δ, jhd1Δ, jhd2Δ*). As shown in Fig. 1a, the top hit candidate of the screening was the HAT mutant *rtt109Δ*, with 37% of the nuclei showing S9.6 signal vs the 17% of the wild-type strain. Since S9.6 signal can be due to dsRNA cross-contamination, we performed DRIP followed by qPCR at 4 genes (*PDC1*, *GCN4*, *SPF1*, and *PDR5*) that had been previously reported as hybrid-prone (Castellano-Pozo *et al.* 2013; Garcia-Pichardo *et al.* 2017; Lafuente-Barquero *et al.* 2017), and by in vitro treatment with RNH1, which specifically degrades the RNA within DNA–RNA hybrids prior to IP (Fig. 1b). We observed that *RTT109* loss increased DNA–RNA hybrids at the 4 genes, the increase being statistically significant for *PDC1*, *SPF1*, and *PDR5* (Fig. 1b). Importantly, RNH1 treatment significantly reduced the S9.6 signal detected at all regions confirming that it was specific for DNA–RNA hybrids.

To test whether the accumulation of DNA–RNA hybrids in *rtt109Δ* HAT-deficient cells was a consequence of the inability to acetylate its targets, we studied S9.6 signals in *rtt109Δ* cells transformed with a plasmid containing either a wild-type *RTT109* or the *rtt109-D89A* catalytic mutant (Han, Zhou, Horazdovsky, *et al.* 2007). Whereas wild-type *RTT109* suppressed the increased S9.6 signal, cells transformed with the catalytic mutant showed a significant increase in S9.6 signal indicating that Rtt109 prevents DNA–RNA hybrid accumulation through its catalytic activity (Fig. 1c). Similar results were obtained by DRIP analysis, in which the sensitivity to RNase H confirmed the specificity of the S9.6 antibody for DNA–RNA hybrids (Fig. 1d). Altogether, these results place Rtt109 HAT as a new chromatin factor involved in the homeostasis of DNA–RNA hybrids.

### H3K14A and H3K23A, but not H3K56A, mutations in Rtt109 target residues lead to DNA–RNA hybrid accumulation

**Fig. 2.**
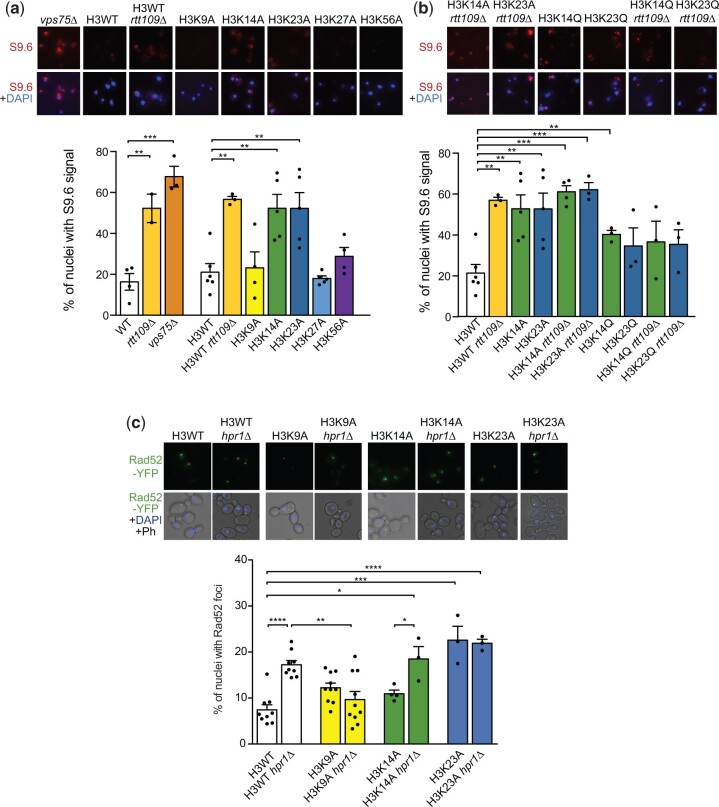
Study of DNA–RNA hybrid accumulation in histone mutations in Rtt109 target residues. a) Percentage of nuclei with S9.6 signal in WT, *rtt109Δ*, *vps75Δ*, H3WT, H3WT *rtt109Δ*, H3K9A, H3K14A, H3K23A, H3K27A, and H3K56A strains. Average and SEM of 2–6 independent experiments and representative images are shown **P < 0.01, ***P < 0.001 (unpaired Student’s *t*-test). b) Percentage of nuclei with S9.6 signal in H3K14A, H3K23A, and acetyl-mimetic H3K14Q and H3K23Q single mutations or in combination with *rtt109Δ*. Average and SEM of 3–6 independent experiments and representative images are shown **P < 0.01, ***P < 0.001 (unpaired Student’s *t*-test). c) Percentage of nuclei with Rad52 foci in H3WT, H3K9A, H3K14A, and H3K23A strains alone or in combination with *hpr1Δ*. Average and SEM of 3–10 independent experiments and representative images are shown. *P < 0.05 (unpaired Student’s *t*-test).

The genetic instability of *rtt109Δ* has been attributed to the loss of acetylation of H3K56, the best-studied target of Rtt109 (Maas *et al.* 2006; Driscoll *et al.* 2007; Han, Zhou, Horazdovsky, *et al.* 2007). Thus, and given that H3K56 acetylation regulates gene expression during DNA replication (Voichek *et al.* 2016), it was possible that deregulated gene expression explained the increased levels of DNA–RNA hybrids of *rtt109Δ.* However, we observed that the DNA–RNA hybrid accumulation was similar in all cell cycle phases upon Rtt109 loss (Supplementary Fig. 1a), which argues against such a possibility. We therefore assessed directly a possible role of H3K56 in R-loop homeostasis by measuring S9.6 signals in the nonacetylable H3K56A histone mutant. For this purpose, we used yeast cells in which one of the genes encoding histone H3 and H4 (*hht1-hhf1*) was deleted, and the other one (*hht2-hhf2*) had either a mutant allele or the wild-type (H3WT strain; Dai *et al.* 2008). DNA–RNA hybrid levels, as determined by either chromosome spreads (Fig. 2a) or DRIP (Supplementary Fig. 1b), were similar in both H3K56A mutant and wild-type H3K56 (H3WT). Moreover, we observed that the deletion of *VPS75*, a histone chaperone that stimulates Rtt109 HAT activity but that is not required for H3K56 acetylation (Han, Zhou, Li, *et al.* 2007), also led to increased S9.6 signal accumulation (Fig. 2a). Altogether, these results rule out H3K56 as the Rtt109 HAT target responsible for R-loop accumulation.

We extended our analysis to other Rtt109 putative targets including H3K9, H3K14, H3K23, and H3K27, reported to be acetylated by Rtt109 in vivo or in vitro (Berndsen *et al.* 2008; Berndsen and Denu 2008; Fillingham *et al.* 2008; Burgess *et al.* 2010). The S9.6 signal detected in H3K9A and H3K27A cells was similar to wild-type levels but, interestingly, both H3K14A and H3K23A mutants showed high levels of S9.6 signal, which were comparable to those of *rtt109Δ* cells (Fig. 2a). Thus, H3K14 and H3K23 could be the Rtt109 targets involved in R-loop homeostasis. This hypothesis predicts that *rtt109Δ* should be epistatic with H3K14A and H3K23A mutations and that the loss of Rtt109 should have no effect in H3K14Q and H3K23Q acetyl-mimetic mutants. Thus, we tested the effect of deleting *RTT109* in these mutant backgrounds. In agreement with our hypothesis, we observed that *rtt109Δ* H3K14A and *rtt109Δ* H3K23A had a similar percentage of cells with S9.6 signal than either of the single mutants (Fig. 2b). Moreover, although H3K14Q and H3K23Q increased the percentage of cells with S9.6 signal, the loss of Rtt109 caused no further effect (Fig. 2b). These results support the view that DNA–RNA hybrid accumulation caused by the loss of Rtt109 is mediated by acetylation of the H3K14 and H3K23 residues.

It is worth noting that R-loop accumulation was previously reported in the full deletion of the histone H3 tail (H3Δ1–28) and the quadruple mutant H3K9-23A (Garcia-Pichardo *et al.* 2017), which contains not only the H3K14A and H3K23A substitutions just shown to accumulate R-loops but also H3K9A and H3K18A substitutions. Notably, these complex mutations of the histone H3 N-terminal tail (H3Δ1-28 and H3K9-23A) prevented the ability of potentially harmful DNA–RNA hybrids, such as those accumulated in the *hpr1Δ* mutant from the THO complex, to cause genetic instability measured as Rad52 foci (Garcia-Pichardo *et al.* 2017). We therefore assayed the ability of H3K9A, H3K14A, and H3K23A to independently suppress the increase in Rad52 foci caused by the *hpr1Δ* mutation (Gavalda *et al.* 2016; Lafuente-Barquero *et al.* 2020). As shown in Fig. 2c, only the H3K9A mutation significantly suppressed this phenotype. Hence, we can now separate the 2 phenotypes of H3K9-23A corresponding to the 2 steps previously shown to be involved in R-loop-associated genetic instability (Garcia-Pichardo *et al.* 2017). Whereas H3K9A prevents the potential harmfulness of hybrids, H3K14A and H3K23A mutations cause the accumulation of DNA–RNA hybrids due to the inability of these mutant forms to be acetylated by Rtt109.

### Changes in the pattern of nucleosome positioning do not correlate with DNA–RNA hybrids

**Fig. 3.**
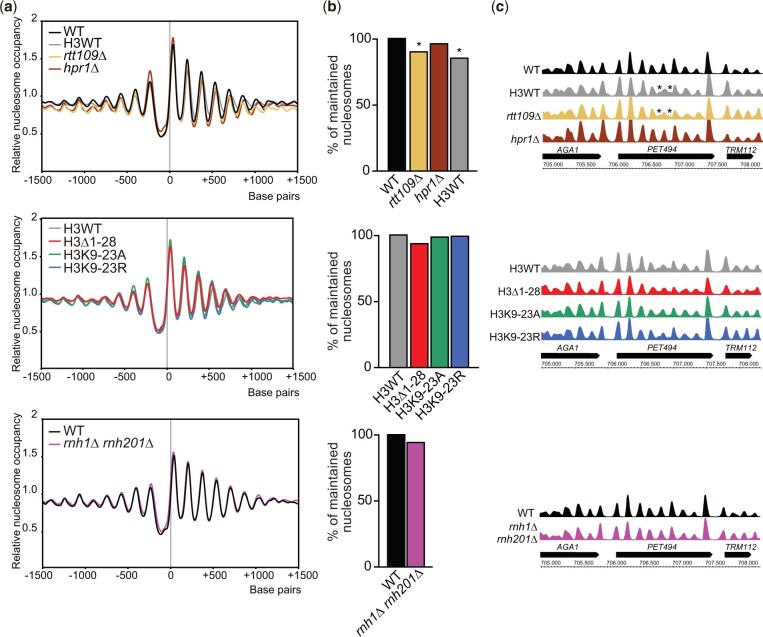
Genome-wide analysis of nucleosome positioning in *rtt109Δ* and histone mutants accumulating R-loops. a) Aggregated profiles of nucleosome occupancy of the indicated strains along 3 kb aligned to the transcription start site (TSS) of 6,572 genes. Position 0 in the *x*-axis indicates the transcription initiation site. b) Danpos comparison of nucleosomal positioning across the genome of the indicated strains. In total, 9.63% and 14.45% of all nucleosomes lost positioning in the *rtt109Δ* and H3WT background relative to the WT BY4741 strain. *P < 0.05 (Fisher test). In all the remaining cases, differences fell within the experimental variation among biological replicates. c) Example of loss of positioning of 2 individual nucleosomes (indicated with an asterisk) in the central region of the *PET494* gene in the *rtt109Δ* and H3WT strains (top panel) and nucleosome mapping in this region in all the indicated strains.

Since Rtt109 is involved in replication-coupled nucleosome assembly (Masumoto *et al.* 2005), and histone mutations can alter nucleosome structure (Dai *et al.* 2008; van Bakel *et al.* 2013), we wondered if putative alterations in nucleosome positioning could cause the accumulation of DNA–RNA hybrids in *rtt109Δ* cells. We performed MNase-Seq analysis in *rtt109Δ* plus the R-loop-positive controls *hpr1Δ* and *rnh1Δ rnh201Δ* and in H3Δ1-28 and H3K9-23A R-loop accumulating histone mutant strains plus the H3K9-23R histone mutant, which does not lead to R-loop accumulation (Garcia-Pichardo *et al.* 2017). Genome-wide comparison of nucleosome positioning with respect to each isogenic wild-type strain by MNase-Seq using Danpos (see *Materials and Methods*) showed that low histone gene dosage had an impact on nucleosome positioning, so that 14.4% of the nucleosomes were differently positioned in the H3WT control with respect to each isogenic control strain (Fig. 3, a and b). More importantly, 9.6% of the nucleosomes lost their positioning in *rtt109Δ* (Fig. 3, a and b) in agreement with its reported role in replication-coupled nucleosome assembly (Masumoto *et al.* 2005). However, these differences were subtle and localized to specific positions in some genes (Fig. 3c). Moreover, no significant changes in nucleosome positioning were observed in either the R-loop-accumulating strains (H3K9-23A, H3Δ1-28, *hpr1Δ* and *rnh1Δ rnh201Δ*) or the R-loop nonaccumulating H3K9-23R mutant with respect to their respective isogenic wild-type backgrounds (Fig. 3, a and b). These results indicate that changes in the pattern of nucleosome positioning are neither the cause nor the consequence of DNA–RNA hybrids or their associated genetic instability.

### DNA–RNA hybrids and DSBs independently contribute to the genetic instability caused by Rtt109 loss

**Fig. 4.**
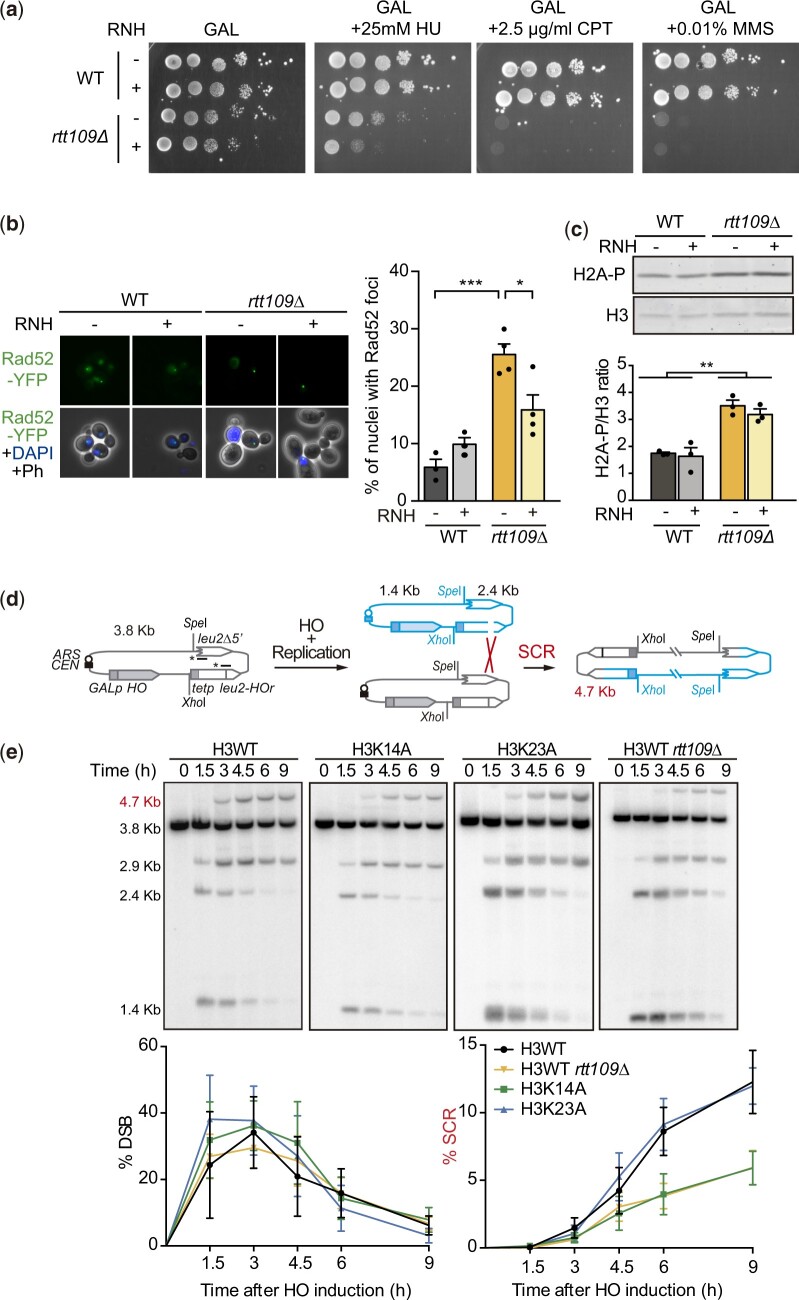
DNA–RNA hybrids and unrepaired DSBs independently contribute to the genetic instability caused by Rtt109 loss. a) Effect of RNase H overexpression on the sensitivity to replication stress. 10-fold serial-dilution assays of WT and *rtt109Δ* transformed with and empty or *GAL::RNH* containing plasmid on minimum complete SGal medium (GAL) with or without HU, MMS, or CPT as indicated. b) Effect of RNase H overexpression on the percentage of nuclei with Rad52 foci in WT and *rtt109Δ* cells. Average and SEM of 3 or 4 independent experiments and representative images are shown. *P < 0.05, ***P < 0.001 (unpaired Student’s *t*-test). c) Representative images and quantification of western blot against global H2A-P levels vs the total amount of histone H3 in WT and *rtt109Δ* transformed with and empty or *GAL::RNH* containing plasmid. Average and SEM of 3 independent experiments are shown **P < 0.01 (unpaired Student’s *t*-test). d) Scheme of the TINV-HO recombination system and SCR intermediates generated after HO-induced replication-born DSBs. DNA was digested with SpeI and XhoI enzymes and SCR intermediates were detected by southern blot hybridization using a radioactive-labeled probe against *LEU2*, depicted with an asterisk. e) Representative gels and quantification of the signal corresponding to the 2.4 and 1.4-kb bands that result from DSB and the 4.7-kb band that correspond specifically to the SCR intermediates during time-course experiments at each of the indicated time points (0, 1.5, 3, 4.5, 6, and 9 h) after galactose addition for HO induction in H3WT, H3K14A, H3K23A, and *rtt109Δ* strains. The 2.9-kb band was not used for the quantification as it can also arise by another non-SCR recombination intermediates. Average and SEM of 3 independent experiments are shown.

Given that DNA–RNA hybrids are a source of recombinogenic DNA damage (Huertas and Aguilera 2003), we wondered whether they contributed to the sensitivity to genotoxic stress or the increased genetic instability previously reported for *rtt109Δ* cells and detected as increased Rad52 foci accumulation (Driscoll *et al.* 2007; Han, Zhou, Horazdovsky, *et al.* 2007). We observed no effect of RNH1 overexpression on the survival of *rtt109Δ* cells to hydroxyurea (HU), methyl methanesulfonate (MMS), or camptothecin (CPT) treatments (Fig. 4a), but RNH1 overexpression in *rtt109Δ* cells partially suppressed the increase in the number of Rad52 foci-containing cells (Fig. 4b). Thus, DNA–RNA hybrids contribute to the genetic instability caused by Rtt109 loss, likely by affecting replication fork progression as shown for other R-loop accumulating mutants. In agreement, *rtt109Δ* is synthetic sick with *rad24Δ*, *rad17Δ* and *ddc1Δ* (Collins *et al.* 2007; Kuzmin *et al.* 2018) similarly to the R-loop accumulating THO mutant *hpr1Δ* (Gomez-Gonzalez *et al.* 2009). However, and in contrast to *hpr1Δ*, *rtt109Δ* is synthetic sick with *rad52Δ* (Pan *et al.* 2006; Alvaro *et al.* 2007; Fillingham *et al.* 2008; Jessulat *et al.* 2008), an indication of DSB accumulation (Tong *et al.* 2004). Indeed, the loss of Rtt109 is known to lead to DSB accumulation, as inferred from the high levels of phosphorylated H2A (H2A-P; Fillingham *et al.* 2008). To test if DNA–RNA hybrids could contribute to the DSBs detected in *rtt109Δ*, we measured the effect of *RNH1* overexpression on H2A-P levels. As shown in Fig. 4c, *RNH1* did not affect H2A-P levels suggesting that DSBs are not induced by hybrids in *rtt109Δ* cells. These results argue in favor of the hypothesis that, as previously proposed for *hpr1Δ* (Gomez-Gonzalez *et al.* 2009), DNA–RNA hybrids do not lead to DSBs directly. We reasoned that replicative stress and checkpoint-deficient conditions might make DNA–RNA hybrids lead to DSBs and render Rad52 essential in *hpr1Δ* cells. Indeed, we observed that *hpr1Δ rad52Δ* cells were extremely sensitive to even the little replicative stress induced by treatment with a very low dose (5 mM) of HU, which depletes the dNTP pools, but only when the checkpoint was compromised (*rad24Δ*, *ddc1Δ*, *rad17Δ*, or *mec3Δ* background; Supplementary Fig. 2a). Therefore, we conclude that whereas DNA–RNA hybrids cause replication fork impairments that can end up in DSBs in the absence of the checkpoint and under further replicative stress conditions, *rtt109Δ* cells not only show DNA–RNA hybrids and associated replication fork impairments but also DSB accumulation that are not caused by R-loops, both phenotypes contributing to the reported increased genetic instability.

### *rtt109Δ* and H3K14A cause inefficient repair of replication-born DSBs, which contributes to replicative stress independently on DNA–RNA hybrids

*rtt109Δ* cells are defective in the repair of replication-born DSBs (Munoz-Galvan *et al.* 2013) that is carried out preferentially by homologous recombination with the sister chromatid (SCR; Kadyk and Hartwell 1992; Johnson and Jasin 2000; Gonzalez-Barrera *et al.* 2003). Thus, DSB accumulation could result from such inefficient repair. Although this defect was attributed to the role of Rtt109 on H3K56 (Munoz-Galvan *et al.* 2013), our new results prompted us to further explore SCR in H3K14A and H3K23A histone mutants. For this purpose, we took advantage of the pTHGH-2 plasmid, which contains both the yeast mating HO endonuclease under the *GAL1* promoter and the TINV-HO recombination system to measure SCR (Ortega *et al.* 2019; Fig. 4d). This system is based on 2 *leu2* inverted-repeats, one of which contains a mini-HO recognition site for the HO endonuclease of 24 bp instead of the total 117 bp (Gonzalez-Barrera *et al.* 2003; Cortes-Ledesma and Aguilera 2006; Ortega *et al.* 2019). Induction of HO expression generates a nick at the mini-HO site that replication would convert into a DSB, which will be preferentially repaired by SCR, whose specific intermediates can be visualized by southern blot (Kadyk and Hartwell 1992; Gonzalez-Barrera *et al.* 2003; Cortes-Ledesma and Aguilera 2006). As shown in Fig. 4e, we confirmed with this assay that DSB induction was similar in all strains and SCR was defective in *rtt109Δ* cells, in agreement with previous results (Munoz-Galvan *et al.* 2013). Interestingly, a similar SCR defect was observed in the H3K14A mutant, but not in H3K23A, indicating that Rtt109 could contribute to SCR not only via acetylation of H3K56 but also by its role on H3K14. Therefore, DSBs accumulate in *rtt109Δ* and H3K14A due to inefficient repair of spontaneous DSBs.

### Rtt109 genetically interacts with Hpr1 upon replicative stress, but not with Sen1

**Fig. 5.**
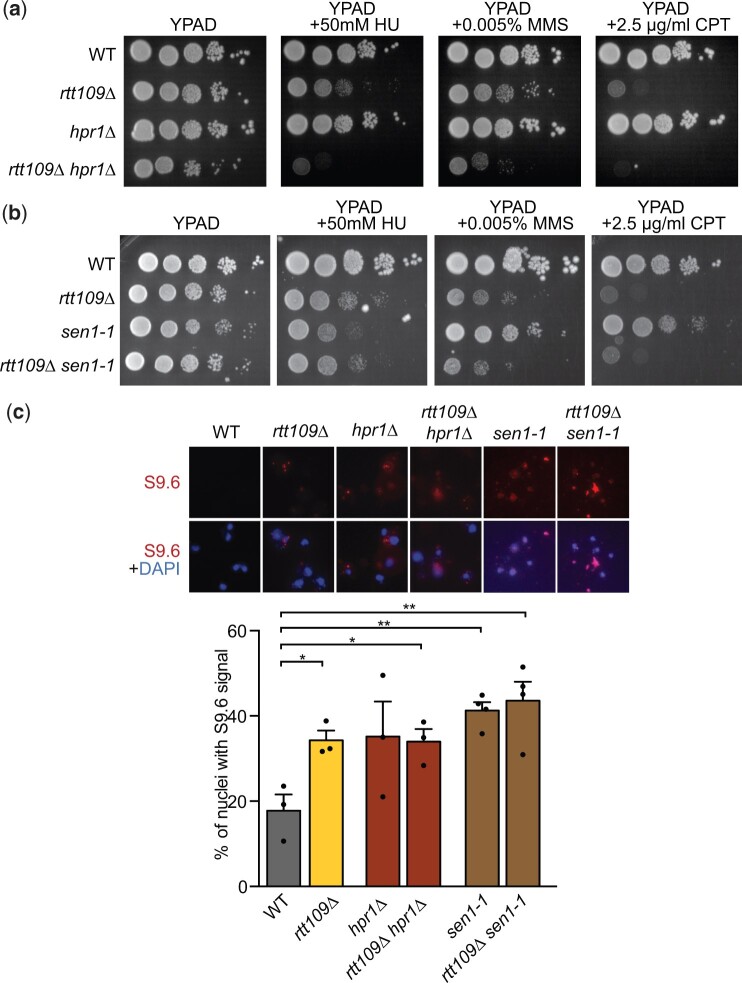
Study of genetic interaction between Rtt109 and Hpr1 or Sen1 in the presence of replicative stress. a) Study of genetic interaction between Rtt109 and Hpr1. Single- and double-mutant sensitivities to replicative stress as determined by 10-fold serial-dilution assays on rich YPAD medium with or without HU, MMS, or CPT as indicated. b) Study of genetic interaction between Rtt109 and Sen1. Single- and double-mutant sensitivities to replicative stress as determined by 10-fold serial-dilution assays on rich YPAD medium with or without HU, MMS, or CPT as indicated. c) Percentage of nuclei with S9.6 signal in WT, *rtt109Δ*, *hpr1Δ*, *rtt109Δ*, *hpr1Δ*, *sen1-1*, and *rtt109Δ sen1-1* strains. Average and SEM of 3 or 4 independent experiments and representative images are shown *P < 0.05, **P < 0.01 (unpaired Student’s *t*-test).

We envision a scenario in which different sources of genetic instability exist in *rtt109Δ* in contrast to R-loop accumulating mutants such as *hpr1Δ*. In *hpr1Δ*, genetic instability is mainly due to the replicative stress caused by the accumulation of cotranscriptional DNA–RNA hybrids. In contrast, both DNA–RNA hybrid accumulation and defective DSB repair independently contribute to the genetic instability caused by Rtt109 loss. Thus, we hypothesized that combining both mutations would have negative consequences by increasing both the occurrence of R-loop-induced DSBs and affecting their repair. In agreement, and as shown in Fig. 5a, *rtt109Δ hpr1Δ* double mutation increased the sensitivity to replicative stress, produced by HU, MMS, or CPT, with respect to either of the single mutants. Notably, this genetic interaction was also observed in H3K14A *hpr1Δ* but not in H3K23A *hpr1Δ* (Supplementary Fig. 2b) suggesting that the enhanced sensitivity of the double mutant was not due to further stimulation of DNA–RNA hybrid formation but to the defect of H3K14A and *rtt109Δ* in DSB repair (Fig. 4e).

We additionally combined *rtt109Δ* with the *sen1-1* mutation in senataxin, which leads to hybrids in S-phase as a consequence of head-on transcription-replication conflicts in contrast to *hpr1Δ*, which leads to cotranscriptional hybrids all throughout the cell cycle (San Martin-Alonso *et al.* 2021). As shown in Fig. 5b, we observed no additive sensitivity to replicative stress in *sen1-1 rtt109Δ* and the levels of S9.6 signal detected were similar in *rtt109Δ sen1-1* or in *rtt109Δ hpr1Δ* and in either of the single mutants (Fig. 5c). These results support that the enhanced sensitivity of *rtt109Δ hpr1Δ* to replicative stress is not just due to further stimulation of DNA–RNA hybrid formation, but to defective repair. Since *rtt109Δ* affects repair via SCR (Munoz-Galvan *et al.* 2013), these data also indicate that *hpr1Δ*-induced fork breakage would be repaired with the sister chromatid whereas *sen1-1*-induced damage would not.

## Discussion

We have uncovered a new role for Rtt109 acetyltransferase in R-loop homeostasis, as *rtt109Δ* mutant causes R-loop accumulation (Fig. 1) due to their incapacity to acetylate H3K14 and H3K23 (Fig. 2). This role of Rtt109 contributes to genetic stability, as the elevated Rad52 foci levels observed upon Rtt109 loss were suppressed after *RNH1* overexpression (Fig. 4). However, this suppression was only partial in contrast to the quasi-full suppression observed in mutants of the THO complex (Gavalda *et al.* 2016; Lafuente-Barquero *et al.* 2020). This is explained by the fact that Rtt109 contributes to genetic stability also by its role in the repair of replication-born DSBs by SCR through the acetylation of H3K56 (Endo *et al.* 2010; Ide *et al.* 2013; Munoz-Galvan *et al.* 2013), and K14, as described here (Fig. 4). Indeed, the DNA damage sensitivity of *rtt109Δ* cells has been attributed to the lack of H3K56 acetylation as H3K56R mutants show sensitivity to HU to the same extent as *rtt109Δ* or *rtt109Δ* H3K56R double mutants (Driscoll *et al.* 2007; Han, Zhou, Horazdovsky, *et al.* 2007). In contrast, the number of cells with Rad52 foci was reported higher in *rtt109Δ* than in H3K56R (Han, Zhou, Horazdovsky, *et al.* 2007) and we observed that RNH1 overexpression partially suppressed this phenotype (Fig. 4), supporting that both the lack of H3K56 acetylation and DNA–RNA hybrids contribute together to the replication stress of *rtt109Δ* cells. Acetylated H3K56 is a mark for newly synthesized chromatin during replication (Masumoto *et al.* 2005; Han, Zhou, Horazdovsky, *et al.* 2007) that has been reported to have a role in SCR due to the asymmetry of the acetylation state between replicated and nonreplicated sequences around the fork (Munoz-Galvan *et al.* 2013). Rtt109 only functions on free nucleosomes (Han, Zhou, Li, *et al.* 2007), so the involvement of H3K14 acetylation in SCR could be caused by a similar mechanism. Indeed, we believe that H3K14 acetylation prevents also hybrid accumulation behind replication forks, as we observed no Rad52 foci accumulation in H3K14A (Fig. 2c), which suggests that the hybrids that are formed in this mutant do not lead to fork stalling. Alternatively, since it has been shown that nucleosomes with acetylated H3K14 have a higher affinity for the purified chromatin remodeling complex RSC (Duan and Smerdon 2014), which is known to facilitate SCR (Oum *et al.* 2011), it is possible that the SCR defect of H3K14A could be due to a deficiency in RSC function.

Our observations nail down the mutations responsible for the R-loop accumulation previously observed in H3K9-23A and H3Δ1-28 (Garcia-Pichardo *et al.* 2017) to the H3K14A and H3K23A mutations, likely due to the lack of acetylation by Rtt109. Interestingly, mass spectrometry and biochemical and genomic analyses have revealed a coexistence of acetylated H3K14 and K23 in human cells (Klein *et al.* 2019) and these histone marks have been linked to oncogenesis (Groner *et al.* 2016; Lv *et al.* 2017), consistent with their relevance in genome instability, a hallmark of cancer cells. Yet, the molecular mechanism to explain R-loop accumulation in these mutated histone contexts remains elusive. Lysine acetylation can act by directly altering the structure of chromatin weakening the electrostatic interactions with DNA and resulting in less compact chromatin. In this sense, histone hyperacetylation caused by the loss of HDACs enhances the formation of R-loops likely by making a more open and accessible chromatin (Salas-Armenteros *et al.* 2017). In contrast, for the R-loop accumulation upon the loss of an HAT, as Rtt109, we favor an indirect model in which the interaction of downstream factors to H3K14 and H3K23 acetylated residues is affected, the nature of these interactions yet to be uncovered.

A recent report has suggested a role for the Rtt109-mediated acetylation of these H3 N-terminal tail residues in the new deposition of nucleosomes ahead of the fork (Frenkel *et al.* 2021). However, we failed to detect a significant correlation between the percentage of misallocated nucleosomes and R-loops (Fig. 3) disregarding the possibility that alterations in nucleosome positioning could relate to the observed R-loop accumulation. Still, since R-loops are rare events occurring at a low frequency, it is unlikely that they cause defects in nucleosome positioning profiles of whole populations. The same study (Frenkel *et al.* 2021) reported that the lack of nucleosome deposition ahead of forks causes faster fork speed in *rtt109Δ*. Such aberrant fast forks might cause the R-loop accumulation possibly by supercoiling alterations. Alternatively, given that DNA–RNA hybrids can be stimulated by DNA breaks [reviewed in Aguilera and Gomez-Gonzalez (2017)], the persistence of unrepaired DSBs (Fig. 4) could explain the accumulation of DNA–RNA hybrids in *rtt109Δ* and H3K14A cells (Figs. 1 and 2). However, we did not detect hybrids in H3K56A mutant (Fig. 2), which is also deficient in repair by SCR (Munoz-Galvan *et al.* 2013), implying that if any, DSB-trapped DNA–RNA hybrids have a poor contribution.

**Fig. 6.**
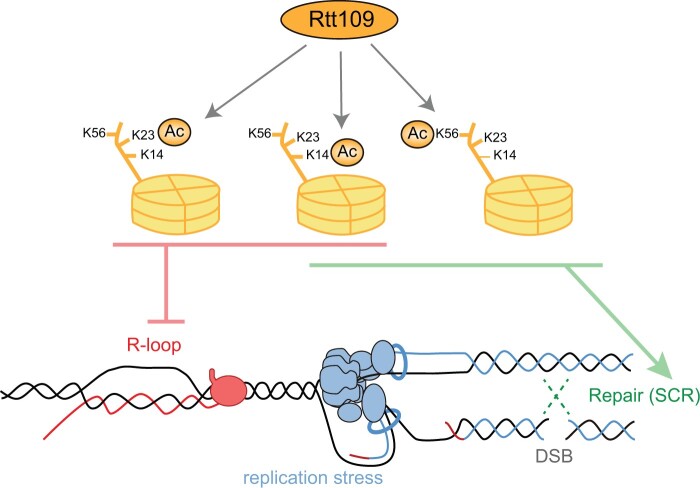
Model for the roles of Rtt109 in R-loop metabolism and associated genetic instability. Rtt109 mediates the acetylation (Ac) of multiple targets, among which the acetylation of H3K14 and H3K23 contribute to prevent R-loops whereas the acetylation of H3K14 and H3K56 is important for repair by SCR. High levels of R-loops cause replication stress that may lead to DSBs that would be preferentially repaired by SCR.

We propose a model (Fig. 6) in which Rtt109 counteracts DNA–RNA hybrid formation by a mechanism that involves H3K23 and H3K14 acetylation. If formed ahead of replication forks, high levels of R-loops cause fork stalling and ultimately lead to fork breakage, particularly in the absence of a proper DNA damage-checkpoint response and under further replicative stress, as shown for *hpr1Δ* (Supplementary Fig. 2). Broken forks would be preferentially repaired with the sister chromatid. Inefficient repair with the sister chromatid, as it happens in *rtt109Δ* or H3K14A that are defective in SCR (Fig. 4), would explain the genetic interaction between Rtt109 and Hpr1 (Fig. 5). In contrast, R-loops that accumulate in association with head-on transcription-replication conflicts, such as those formed in the absence of Sen1, would lead to DSBs ahead of the fork (San Martin-Alonso *et al.* 2021). Therefore, in this case, DSB repair would not be with the sister chromatid, because it is not yet available, so Rtt109 would not be involved.

In conclusion, our results place the Rtt109 acetyltransferase as a complex modulator of R-loops and their associated DNA damage by acetylating multiple histone H3 tail targets and support the conclusion that chromatin is a major determinant in R-loop-associated genetic instability.

## Data availability

Strains and plasmids are available upon request. Sequencing raw reads (FASTQ files) and processed file are available from the Gene Expression Omnibus database (accession number GSE192701). The rest of the relevant data are shown within the manuscript and in the Supplementary File 1.

Supplemental material is available at *GENETICS* online.

## Supplementary Material

iyac108_File_S1

iyac108_Table_S1

iyac108_Table_S2

iyac108_Figure_S1

iyac108_Figure_S2
