# Supplementary material for: A role for the Saccharomyces cerevisiae Rtt109 histone acetyltransferase in R-loop homeostasis and associated genome instability
Source: Genetics. 2022 Jul 22;222(1):iyac108. doi: 10.1093/genetics/iyac108 (PMC9434296; doi:10.1093/genetics/iyac108)
Supplement: iyac108_Table_S1 [file iyac108_table_s1.docx]

Table S1. Yeast strains used in this study.

| **Strain** | **Genotype** | **Source** |
| --- | --- | --- |
| BY4741 | *MATa his3∆1 leu2∆0 met15∆ ura3∆0* | Euroscarf |
| Y02827 | BY4741 *hat1Δ::Kan* | Euroscarf |
| Y00297 | BY4741 *hat2Δ::Kan* | Euroscarf |
| Y04282 | BY4741 *ada2Δ::Kan* | Euroscarf |
| Y07285 | BY4741 *gcn5Δ::Kan* | Euroscarf |
| Y03218 | BY4741 *spt7Δ::Kan* | Euroscarf |
| Y02666 | BY4741 *spt8Δ::Kan* | Euroscarf |
| Y01490 | BY4741 *rtt109Δ::Kan* | Euroscarf |
| Y04196 | BY4741 *eaf1Δ::Kan* | Euroscarf |
| Y01799 | BY4741 *ahc1Δ::Kan* | Euroscarf |
| Y03078 | BY4741 *sas3Δ::Kan* | Euroscarf |
| Y01114 | BY4741 *rpd3Δ::Kan* | Euroscarf |
| Y01695 | BY4741 *sin3Δ::Kan* | Euroscarf |
| Y00849 | BY4741 *sap30Δ::Kan* | Euroscarf |
| Y06209 | BY4741 *rco1Δ::Kan* | Euroscarf |
| Y05347 | BY4741 *hda1Δ::Kan* | Euroscarf |
| Y05487 | BY4741 *hos1Δ::Kan* | Euroscarf |
| Y07046 | BY4741 *set3Δ::Kan* | Euroscarf |
| Y01801 | BY4741 *hst3Δ::Kan* | Euroscarf |
| Y03550 | BY4741 *hst4Δ::Kan* | Euroscarf |
| Hst3/4 | BY4741 *hst3Δ::Kan hst4Δ::Hph* | This study |
| Y03315 | BY4741 *swd3Δ::Kan* | Euroscarf |
| Y01257 | BY4741 *set2Δ::Kan* | Euroscarf |
| Y04031 | BY4741 *gis1Δ::Kan* | Euroscarf |
| Y04276 | BY4741 *dot1Δ::Kan* | Euroscarf |
| Y06165 | BY4741 *rph1Δ::Kan* | Euroscarf |
| Y00186 | BY4741 *jhd1Δ::Kan* | Euroscarf |
| Y06922 | BY4741 *jhd2Δ::Kan* | Euroscarf |
| Y01198 | BY4741 *vps75Δ::Kan* | Euroscarf |
| RNH-R | *MAT***a** *ade2-1 can1-100 his3-11,15 leu2-3,112 lys2 met15 trp1-1 ura3-1 rnh1Δ::Kan rnh201Δ::Kan* | (Lafuente-Barquero *et al*, 2020) |
| H3WT | *MAT***a** *his3Δ200 leu2Δ0 lys2Δ0 trp1Δ63 ura3Δ0 met15Δ0 can1::MFA1pr-HIS3 hht1-hhf1::Nat hht2-hhf2::[H3]-URA3* | (Dai *et al*, 2008) |
| H3RT | H3WT *rtt109Δ::Kan* | This study |
| H3K9A | H3WT *hht2-hhf2::[H3K9A]-URA3* | (Dai *et al.*, 2008) |
| H3K14A | H3WT *hht2-hhf2::[H3K14A]-URA3* | (Dai *et al.*, 2008) |
| H3K23A | H3WT *hht2-hhf2::[H3K23A]-URA3* | (Dai *et al.*, 2008) |
| H3K27A | H3WT *hht2-hhf2::[H3K27A]-URA3* | (Dai *et al.*, 2008) |
| H3K56A | H3WT *hht2-hhf2::[H3K56A]-URA3* | (Dai *et al.*, 2008) |
| H3K14Q | H3WT *hht2-hhf2::[H3K14Q]-URA3* | (Dai *et al.*, 2008) |
| H3K23Q | H3WT *hht2-hhf2::[H3K23Q]-URA3* | (Dai *et al.*, 2008) |
| H3K14QRt | H3K14Q *rtt109Δ::Kan* | This study |
| H3K23QRt | H3K23Q *rtt109Δ::Kan* | This study |
| H3HP1 | H3WT *hpr1Δ::Hph* | This study |
| H3K9AHP | H3K9A *hpr1::Kan* | This study |
| H3K14ARt | H3K14A *rtt109Δ::Kan* | This study |
| H3K23ARt | H3K23A *rtt109Δ::Kan* | This study |
| H3K14AHP | H3K14A *hpr1Δ::Kan* | This study |
| H3K23AHP | H3K23A *hpr1Δ::Kan* | This study |
| Y04072 | BY4741 *hpr1Δ::Kan* | Euroscarf |
| H3∆1-28 | *MAT***a** *his3Δ200 leu2Δ0 lys2Δ0 trp1Δ63 ura3Δ0 met15Δ0 can1::MFA1pr-HIS3 hht1-hhf1::Nat hht2-hhf2::[∆1-28]-URA3* | (Dai *et al.*, 2008) |
| H3K9-23A | *MAT***a** *his3Δ200 leu2Δ0 lys2Δ0 trp1Δ63 ura3Δ0 met15Δ0 can1::MFA1pr-HIS3 hht1-hhf1::Nat hht2-hhf2::[K9-23A]-URA3* | (Dai *et al.*, 2008) |
| H3K9-23R | *MAT***a** *his3Δ200 leu2Δ0 lys2Δ0 trp1Δ63 ura3Δ0 met15Δ0 can1::MFA1pr-HIS3 hht1-hhf1::Nat hht2-hhf2::[K9-23R]-URA3* | (Dai *et al.*, 2008) |
| BYRtH | *MAT***a** *his3Δ1 leu2Δ0 met15Δ0 ura3Δ0 rtt109Δ::Nat hpr1Δ::Kan* | This study |
| W303-1A | *MAT***a** *ade2-1 can1-100 his3-11,15 leu2-3,112 trp1-1 ura3-1* |  |
| SEN1-R | W303-1A RAD5 *bar1∆ sen1-1* | (San Martin-Alonso *al.,* 2021) |
| BYRtS | W303-1A RAD5 *bar1∆ sen1-1 rtt109∆Kan* | This study |
| U678-1C | W303-1A *hpr1Δ::HIS3* | (Piruat & Aguilera, 1998) |
| WR24-6C | W303-1B *rad24Δ::TRP1* | (Gomez-Gonzalez *et al*, 2009) |
| WHR24-3B | W303-1A *hpr1Δ::HIS3 rad24Δ::TRP1* | (Gomez-Gonzalez *et al.*, 2009) |
| WSR52-4B | W303-1A *Mat a-inc leu2∆::SFA rad52Δ::KanMX rad24Δ::TRP1* | (Gonzalez-Barrera *et al*, 2003) |
| WHR52-1A | W303-1A *hpr1Δ::HIS3 rad52Δ::KanMX4* | (Gomez-Gonzalez *et al.*, 2009) |
| R24R52-1D | W303-1A *rad24Δ::TRP1 rad52Δ::KanMX4* | This study |
| WHRR-18D | W303-1A *hpr1Δ::HIS3 rad24Δ::TRP1 rad52Δ::KanMX4* | This study |
| YLL244 | W303-1A *ddc1Δ::KanMX4* | (Majka & Burgers, 2005) |
| HDDC1-4B | W303-1A *hpr1Δ::HIS3 ddc1Δ::KanMX4* | (Gomez-Gonzalez *et al.*, 2009) |
| YBG723 | W303-1A *hpr1Δ::HIS3 ddc1Δ::KanMX4 rad52Δ::KanMX4* | This study |
| YLL134 | W303-1A *mec3Δ::TRP1* | (Majka & Burgers, 2005) |
| HMEC3-1A | W303-1A *hpr1Δ::HIS3 mec3Δ::TRP1* | (Gomez-Gonzalez *et al.*, 2009) |
| YBG725 | W303-1A *hpr1Δ::HIS3 mec3Δ::TRP1 rad52Δ::KanMX4* | This study |
| DMP1913-11C | W303-1A *rad17Δ::LEU2* | (Majka & Burgers, 2005) |
| HRAD17-9D | W303-1A *hpr1Δ::HIS3 rad17Δ::LEU2* | (Gomez-Gonzalez *et al.*, 2009) |
| YBG727 | W303-1A *hpr1Δ::HIS3 rad17Δ::LEU2 rad52Δ::KanMX4* | This study |

Dai J, Hyland EM, Yuan DS, Huang H, Bader JS, Boeke JD (2008) Probing nucleosome function: a highly versatile library of synthetic histone H3 and H4 mutants. *Cell* 134: 1066-1078

Gomez-Gonzalez B, Felipe-Abrio I, Aguilera A (2009) The S-phase checkpoint is required to respond to R-loops accumulated in THO mutants. *Mol Cell Biol* 29: 5203-5213

Gonzalez-Barrera S, Cortes-Ledesma F, Wellinger RE, Aguilera A (2003) Equal sister chromatid exchange is a major mechanism of double-strand break repair in yeast. *Mol Cell* 11: 1661-1671

Lafuente-Barquero J, Garcia-Rubio ML, Martin-Alonso MS, Gomez-Gonzalez B, Aguilera A (2020) Harmful DNA:RNA hybrids are formed in cis and in a Rad51-independent manner. *Elife* 9

Majka J, Burgers PM (2005) Function of Rad17/Mec3/Ddc1 and its partial complexes in the DNA damage checkpoint. *DNA Repair (Amst)* 4: 1189-1194

Piruat JI, Aguilera A (1998) A novel yeast gene, THO2, is involved in RNA pol II transcription and provides new evidence for transcriptional elongation-associated recombination. *EMBO J* 17: 4859-4872

San Martin-Alonso M, Soler-Oliva ME, García-Rubio M, García-Muse, T,Aguilera A. (2021). Harmful R-loops are prevented via different cell cycle-specific mechanisms. *Nature communications*, *12*(1), 4451.
