# Supplementary material for: A role for the Saccharomyces cerevisiae Rtt109 histone acetyltransferase in R-loop homeostasis and associated genome instability
Source: Genetics. 2022 Jul 22;222(1):iyac108. doi: 10.1093/genetics/iyac108 (PMC9434296; doi:10.1093/genetics/iyac108)
Supplement: iyac108_Table_S2 [file iyac108_table_s2.docx]

Table S2. Plasmids used in this study.

| **Plasmid** | **Description** | **Source** |
| --- | --- | --- |
| pWJ1213 | YCp containing RAD52::YFP fusion under its own promoter | (Lisby *et al*, 2001) |
| pWJ1344 | YCp containing RAD52::YFP fusion under its own promoter | (Lisby *et al.*, 2001) |
| pRS317GAL | pRS317 with the GAL1 promoter and the CYC1-terminator | (Eriksson *et al*, 2004) |
| Rtt109 WT | pRS316 with wild-type *RTT109* gene | (Han *et al*, 2007) |
| D89A | pRS316 with *RTT109* gene mutated at aspartate 89 to alanine | (Han *et al.*, 2007) |
| pRS317-GAL::RNH1 | pRS317 containing the GALp::RNH1 fusion | (Garcia-Pichardo *et al*, 2017) |
| pRS314 | YCp plasmid with *TRP1* as a marker | (Sikorski & Hieter, 1989) |
| pRS315 | YCp plasmid with *LEU2* as a marker | (Sikorski & Hieter, 1989) |
| pRS316 | YCp plasmid with *URA3* as a marker | (Sikorski & Hieter, 1989) |
| pRS317 | YCp plasmid with *LYS2* as a marker | (Eriksson *et al*, 2004) |
| pRS315-GAL::RNH1 | pRS315 containing the GALp::RNH1 fusion | (Gomez-Gonzalez *et al*, 2011) |
| pTHGH-2 | YCp plasmid with *TRP1* as a marker with the TINV recombination system and GAL::HO | (Ortega *et al*, 2019) |

Eriksson P, Thomas LR, Thorburn A, Stillman DJ (2004) pRS yeast vectors with a LYS2 marker. *Biotechniques* 36: 212-213

Garcia-Pichardo D, Canas JC, Garcia-Rubio ML, Gomez-Gonzalez B, Rondon AG, Aguilera A (2017) Histone Mutants Separate R Loop Formation from Genome Instability Induction. *Mol Cell* 66: 597-609 e595

Gomez-Gonzalez B, Garcia-Rubio M, Bermejo R, Gaillard H, Shirahige K, Marin A, Foiani M, Aguilera A (2011) Genome-wide function of THO/TREX in active genes prevents R-loop-dependent replication obstacles. *EMBO J* 30: 3106-3119

Han J, Zhou H, Horazdovsky B, Zhang K, Xu RM, Zhang Z (2007) Rtt109 acetylates histone H3 lysine 56 and functions in DNA replication. *Science* 315: 653-655

Lisby M, Rothstein R, Mortensen UH (2001) Rad52 forms DNA repair and recombination centers during S phase. *Proc Natl Acad Sci U S A* 98: 8276-8282

Ortega P, Gomez-Gonzalez B, Aguilera A (2019) Rpd3L and Hda1 histone deacetylases facilitate repair of broken forks by promoting sister chromatid cohesion. *Nat Commun* 10: 5178

Sikorski RS, Hieter P (1989) A system of shuttle vectors and yeast host strains designed for efficient manipulation of DNA in Saccharomyces cerevisiae. *Genetics* 122: 19-27
