# Supplementary figures and images for: A role for the Saccharomyces cerevisiae Rtt109 histone acetyltransferase in R-loop homeostasis and associated genome instability
Source: Genetics. 2022 Jul 22;222(1):iyac108. doi: 10.1093/genetics/iyac108 (PMC9434296; doi:10.1093/genetics/iyac108)

**A**

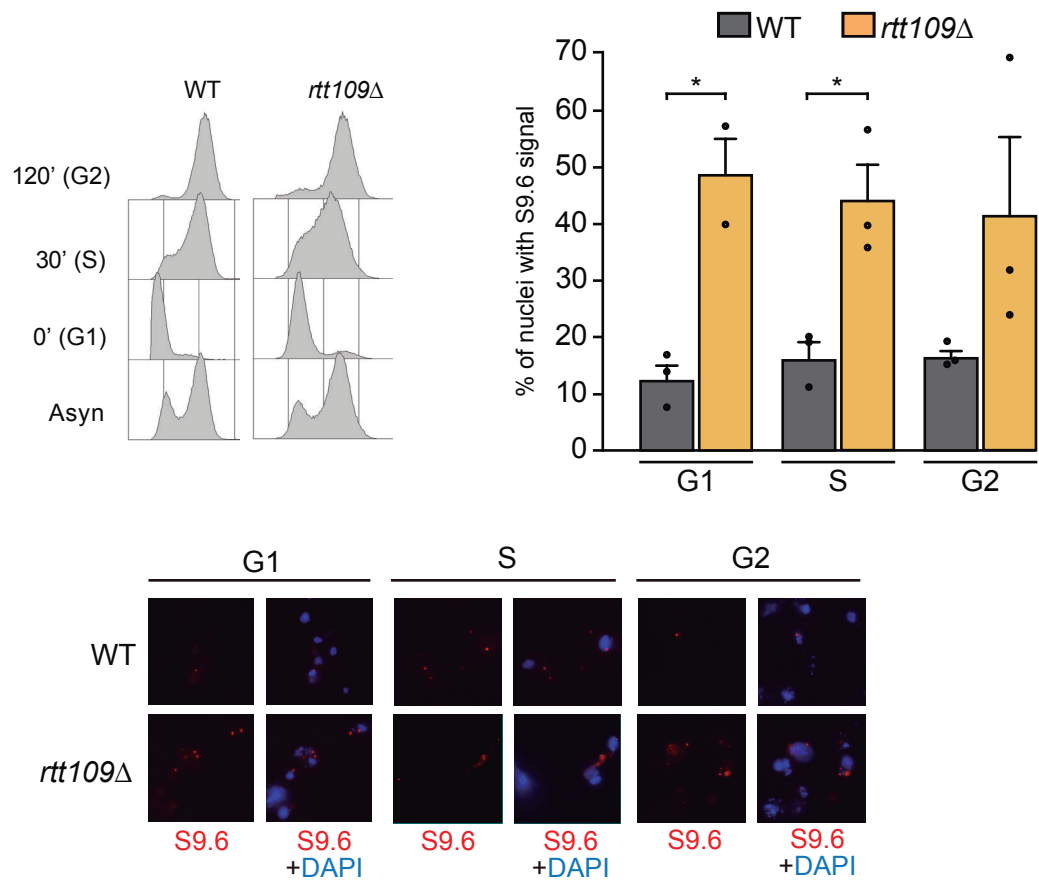

**B**

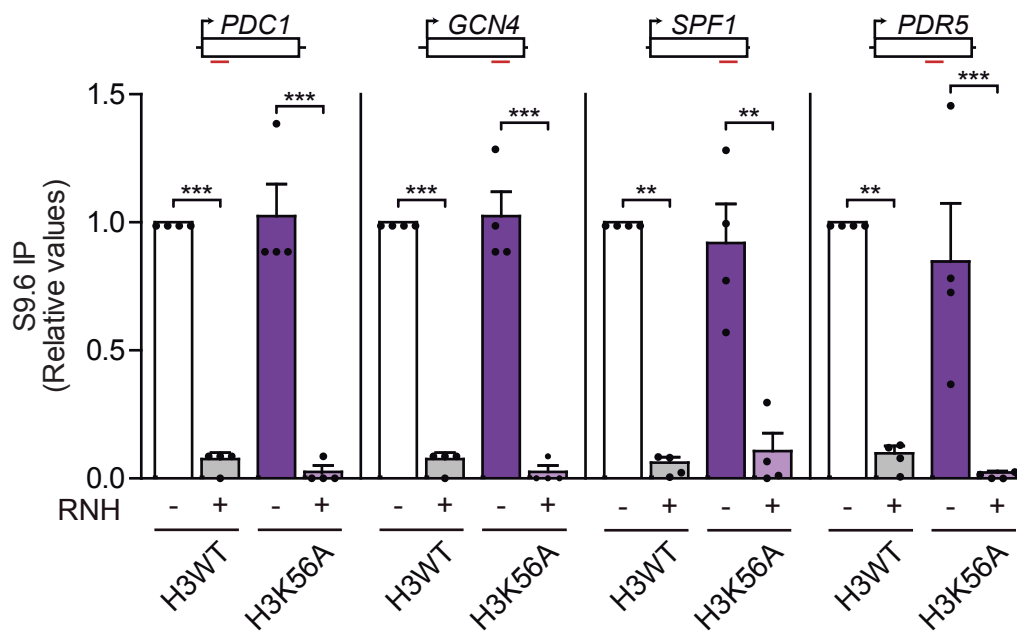

Supplement: iyac108_Figure_S1 [file iyac108_figure_s1.pdf]

**A**

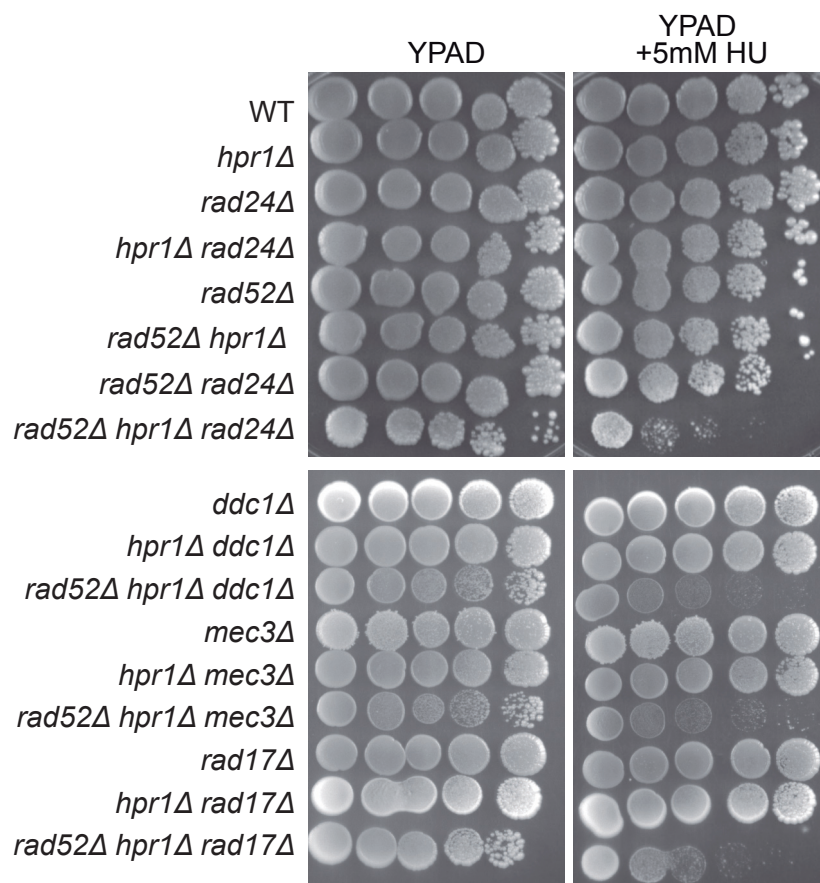

**B**

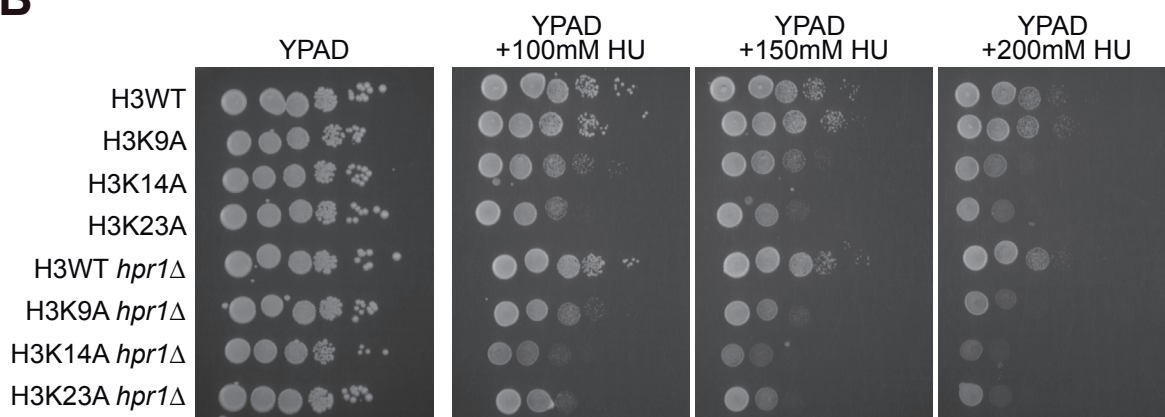

Supplement: iyac108_Figure_S2 [file iyac108_figure_s2.pdf]
